# PERSPECTIVES OF THE FEMALE SPOUSE IN DEMENTIA CAREGIVING IN FORMAL CAREGIVING RESOURCE USE

**DOI:** 10.1093/geroni/igac059.3043

**Published:** 2022-12-20

**Authors:** Anna Satake

**Affiliations:** Univerisity of California, Davis, American Canyon, California, United States

## Abstract

The purpose of this qualitative descriptive study was to explore female spousal caregivers’ decisions regarding the use of formal caregiving resources in caring for a partner with dementia. Participants included 11 female spouses who provided caregiving to their husbands with dementia in the home setting. Findings suggested that a caregiver’s willingness or reluctance to use any resources were influenced by factors that related to them (e.g., assessment of their skills/abilities), their husband (e.g., his belief he didn’t need help) and, to a lesser degree, the resource qualities (e.g., cost). Caregivers seemed to make the decision to use resources only after careful consideration of their husband’s needs, his understanding of what he needed, and how well the resource would fit for him and whether he would accept that help. Interestingly, factors like cost and access were less germane to the caregiver’s decision. This implies that the decision-making process for a wife caregiver in regards to the care for a partner with dementia is complex and involves their readiness and needs as the caregiver, the needs and readiness of the persons with dementia, and the qualities of the resources. These factors are all dynamic, and clinicians and formal caregiving services need to have ongoing conversations and assessments throughout the caregiving journey to assess both the caregiver’s willingness and reluctance to start a specific service.

